# GENDERED PATHWAYS TO FINANCIAL CAPABILITY: IMPLICATIONS FOR PHYSICAL, MENTAL, AND FINANCIAL HEALTH IN LATER LIFE

**DOI:** 10.1093/geroni/igad104.1649

**Published:** 2023-12-21

**Authors:** Yu-Chih Chen, Sicong Sun

**Affiliations:** The University of Hong Kong, Hong Kong, Hong Kong; The University of Kansas, Lawrence, Kansas, United States

## Abstract

Financial capability, comprising financial literacy, access, and behavior, can influence an individual’s ability to effectively use financial resources, thus impacting their health and well-being. However, studies have predominantly focused on financial literacy and overlooked a more comprehensive measure of financial capability. Additionally, there is limited knowledge on the role of gender in these associations. This study investigated how gender may moderate the links between financial capability and health. The study recruited 1,109 community-dwelling adults (aged 45+) through an online survey and employed multivariate linear and logistic regression to examine the gender differences in the associations between financial capability and physical (perceived health and mobility limitations), mental (life satisfaction and depression), and financial (retirement worry and financial satisfaction) health. Results showed that financial access and behavior, compared to financial literacy, had a more significant influence on determining health outcomes. Gender differences in financial capability were also identified through simple slope analyses, with financial literacy being more important for men’s self-rated health and life satisfaction, while financial behavior was more critical for women. Additionally, although financial access was not related to retirement worry among men, it was significantly associated with lower retirement worry among women. Findings suggest that gender-specific pathways to financial capability may lead to health disparities. Policies and programs aimed at improving population’s health and well-being, particularly for women, should target not only financial literacy but also strengthen financial inclusion and encourage responsible financial behavior.

